# Antiamoebic Properties of Laboratory and Clinically Used Drugs against *Naegleria* *fowleri* and *Balamuthia mandrillaris*

**DOI:** 10.3390/antibiotics11060749

**Published:** 2022-05-31

**Authors:** Ruqaiyyah Siddiqui, Mohammad Ridwane Mungroo, Tengku Shahrul Anuar, Ahmad M. Alharbi, Hasan Alfahemi, Adel B. Elmoselhi, Naveed Ahmed Khan

**Affiliations:** 1 College of Arts and Sciences, American University of Sharjah, Sharjah 26666, United Arab Emirates; rsiddiqui@aus.edu (R.S.); ridwane@yahoo.com (M.R.M.); 2 Centre for Medical Laboratory Technology, Faculty of Health Sciences, Puncak Alam Campus, Universiti Teknologi MARA, Shah Alam 40450, Selangor, Malaysia; tengku9235@uitm.edu.my; 3 Department of Clinical Laboratory Sciences, College of Applied Medical Sciences, Taif University, Taif 21944, Saudi Arabia; a.alharbii@tu.edu.sa; 4 Department of Medical Microbiology, Faculty of Medicine, Al-Baha University, Al-Baha 65799, Saudi Arabia; halfahmi@bu.edu.sa; 5 Department of Basic Medical Sciences, College of Medicine, University of Sharjah, Sharjah 27272, United Arab Emirates; adel@yhaoo.com; 6 Department of Clinical Sciences, College of Medicine, University of Sharjah, Sharjah 27272, United Arab Emirates

**Keywords:** brain-eating amoeba, *Naegleria fowleri*, *Balamuthia mandrillaris*, amoebicidal, cysticidal, excystation, trophozoites, cysts

## Abstract

*Naegleria* *fowleri* and *Balamuthia mandrillaris* are pathogenic free-living amoebae that infect the central nervous system with over 95% mortality rates. Although several compounds have shown promise in vitro but associated side effects and/or prolonged approval processes for clinical applications have led to limited success. To overcome this, drug repurposing of marketed compounds with known mechanism of action is considered a viable approach that has potential to expedite discovery and application of anti-amoebic compounds. In fact, many of the drugs currently employed in the treatment of *N. fowleri* and *B. mandrillaris*, such as amphotericin B, fluconazole, rifampin and miltefosine, are repurposed drugs. Here, we evaluated a range of clinical and laboratory compounds including metformin, quinclorac, indaziflam, inositol, nateglinide, 2,6-DNBT, trans-cinnamic acid, terbuthylazine, acarbose, glimepiride, vildagliptin, cellulase, thaxtomin A, repaglinide and dimethyl peptidase (IV) inhibitor against *N. fowleri* and *B. mandrillaris*. Anti-amoebic assays revealed that indaziflam, nateglinide, 2,6-DNBT, terbuthylazine, acarbose and glimepiride exhibited potent amoebicidal properties against both *N. fowleri* and *B. mandrillaris*. Notably, all compounds tested showed minimal human (HaCaT) cell cytotoxicity as determined by lactate dehydrogenase release. Prospective research using animal models is warranted to determine the potential of these repurposed compounds, as well as the need for investigating the intranasal route of delivery to treat these devastating infections.

## 1. Introduction

Free-living amoebae, including *Naegleria fowleri* and *Balamuthia mandrillaris* are well-regarded as opportunistic protozoan pathogens which can cause infection of the central nervous system (CNS), that almost always leads to death. In part, this is due to non-availability of effective treatments, incomplete understanding of the pathogenesis as well as high virulence of amoebae [1,2,3,4]. *N. fowleri* infects the CNS of young children and adults, causing primary amoebic meningoencephalitis (PAM) characterized by a rapid onset of disease with symptoms including headache, fever, nausea, or vomiting leading to stiff neck, confusion, lack of attention, loss of balance, seizures, hallucinations and death within days [5]. Likewise, *B. mandrillaris* also targets the CNS causing granulomatous amoebic encephalitis (GAE) with symptoms including headaches, stiff neck or head and neck pain with neck movement, sensitivity to light, nausea, vomiting, lethargy, low-grade fever, mental health changes, seizures, confusion, partial paralysis, difficulty in walking and speaking, seizures leading to death within weeks to months [6]. The mortality associated with both PAM and GAE is very high, resulting in death in over 95% of the reported cases [6,7,8,9]. Furthermore, due to an increase in global warming and the reliance of people on storage water tanks; where these amoebae can thrive, it is anticipated that infections due to these amoebae will rise [10,11]. 

Several compounds have shown promise in vitro but associated side effects and prolonged approval processes for clinical application of compounds together with rarity of the disease has hampered progress in the development of effective treatment. To overcome this, drug repurposing of clinically-used compounds with known mode of action is considered a viable approach that has potential to expedite discovery and application of anti-amoebic compounds [12,13]. Given that glucose is an important constituent in the cyst wall composition and/or its biosynthesis, we evaluated a range of clinical and non-clinical compounds including metformin, quinclorac, indaziflam, inositol, nateglinide, 2,6-DNBT, trans-cinnamic acid, terbuthylazine, acarbose, glimepiride, vildagliptin, cellulase, thaxtomin A, repaglinide and dimethyl peptidase(iv) inhibitor [13,14,15,16,17,18,19,20]. These were selected as they showed previous activity against other microbial pathogens. By employing bioassay-guided investigation with aforementioned compounds, amoebicidal effects, and anti-excystation properties as well as human cell cytotoxicity was determined as described previously [21,22]. These studies are important in order to develop much needed compounds for these devastating infections, and they should be evaluated in both vaporised formulations to test intranasally, as well as in the traditional intravenous treatment form.

## 2. Materials and Methods

### 2.1. Henrietta Lacks (HeLa) and HaCaT Cell Culture

Human cervical adenocarcinoma (HeLa) cells (ATCC^®^ CCL-2^™^) were purchased from American Type Culture Collection, while human keratinized skin (HaCaT) cells (CLS:300493) were acquired from CLS Cell Lines. Cells were cultivated in Roswell Park Memorial Institute (RPMI) 1640 medium, supplemented with 10% foetal bovine serum (FBS), 1% L-glutamine, 1% antibiotics and 1% minimum essential medium amino acids at 37 °C and 5% CO_2_ in a humidified incubator as previously described [23].

### 2.2. Culture of Amoebae

*N. fowleri* cells isolated from the cerebrospinal fluid of a patient (ATCC 30174) and *B. mandrillaris* cells from the brain of a mandrill baboon (ATCC 50209) were utilized in this study. Amoebae were cultured as previously described [24,25]. Briefly, the amoebae were cultured in RPMI-1640 medium supplemented with 1% antibiotics with HeLa cell monolayers as food source at 37 °C in a 5% CO_2_ incubator. Once the HeLa cell monolayers are depleted, the amoebae are collected through centrifugation and transferred into new flasks with HeLa cell monolayers as food source or into flasks without cell monolayers to trigger the formation of cysts.

### 2.3. Chemicals

Compounds were purchased and dissolved in RPMI-1640, 20% ethanol or 20% DMSO. For ethanol and DMSO, compounds were first dissolved in 100% ethanol or 100% DMSO (e.g., 200 µL). Next, these solutions were diluted in RPMI-1640 (e.g., 800 µL) to obtain 5 mM final stock concentration of the compound. For negative controls, ethanol or DMSO alone were mixed with RPMI-1640 using relative amounts. Table 1 depicts the chemical nomenclature, empirical formula, class, mechanism of action, molar mass and solvent solubility of the compounds. Stock solutions were prepared at 5 mM concentration.

### 2.4. Amoebicidal Assays

Amoebicidal assays were performed as previously described [26]. Briefly, 5 × 10^5^ amoebae (*N. fowleri* and *B. mandrillaris* cells) were incubated with drugs and/or solvents in 24-well plates in RPMI-1640. Amoebae were incubated with RPMI-1640 for negative controls while DMSO and ethanol were used as solvent control. For the positive control, the anti-amoebic drug, 100 μM miltefosine was used. Plates were incubated at 37 °C and 5% CO_2_ for 24 h. Next, 0.1% Trypan blue was added to each well. Trypan blue is only taken up by dead cells while live cells do not take up the dye and remain unstained. The number of live cells (i.e., unstained) was counted using a haemocytometer to determine amoebicidal effects of various compounds. The data are representative of the mean ± standard error of several experiments performed in duplicate.

### 2.5. Excystation Assays

Briefly, 5 × 10^5^ amoebae cysts (*N. fowleri* and *B. mandrillaris* cells) were incubated with 100 μM drugs and/or solvent in 24-well plates containing RPMI-1640 on HeLa monolayers at 37 °C and 5% CO_2_ until around 75% of the cysts in RPMI-1640 alone have excysted. The RPMI-1640 alone was used as a negative control while DMSO and ethanol were used as a solvent control and 100 μM miltefosine was used as positive control. Live trophozoites (amoebae that excysted) were then quantified by counting live (unstained) cells, after addition of 0.1% Trypan blue to each well, using a haemocytometer. Percentage inhibition is defined as: [(Number of trophozoites in untreated well − Number of trophozoites in treated well)/Number of trophozoites in untreated well)] × 100. The data are representative of the mean ± standard error of several independent experiments performed in duplicate.

### 2.6. Cytotoxicity Assays

Cytotoxic effects of the drugs were determined as previously described^21^. Briefly, the cytotoxic activity of the drugs was investigated against HaCaT cells. Drugs were incubated with HaCaT cells in RPMI-1640 for 24 h at 37 °C in a 5% CO_2_ incubator. The RPMI-1640 was then collected and tested for the presence of lactate dehydrogenase (LDH) enzyme by using a cytotoxicity detection kit (Roche Applied Science, New York, NY, USA). The LDH assay protocol is based on an enzymatic reaction, i.e., LDH released from the cell oxidizes lactate to generate NADH, which then reacts with water-soluble tetrazolium salt (WST) to generate a yellow color. The intensity of the generated color correlates directly with the number of lyzed cells. Following this, 1% Triton X-100 was added to the positive control well and incubated the plate for 60 min at 37 °C. For negative control, human cells were grown in RPMI alone well. Then, an equal amount of cells supernatant (comprising LDH enzyme) from each well was combined with equal amount of LDH kit reagents and cytotoxicity was determined in proportion to LDH released from human cells using a spectrophotometer at 490 nm. The percentage LDH released was determined as follows: [(Absorbance of sample − Absorbance of control)/(Absorbance for total LDH release − Absorbance of control)] × 100 = percentage cytotoxicity. 

## 3. Results and Discussion

It is distressing that the mortality rates linked to *Naegleria fowleri* and *Balamuthia mandrillaris* have remained significantly high despite advances in antimicrobial chemotherapy and supportive care. Given the rise in global temperatures and the increasing dependence of the public on water storage tanks where these amoebae thrive, especially in developing countries; it is likely that the number of infections due to these amoebae will increase, thus it is imperative to promptly develop effective treatments against these devastating and fatal infections. Considering that many of the drugs currently employed in the treatment of *N. fowleri* and *B. mandrillaris*, such as amphotericin B, fluconazole, rifampin and miltefosine, are repurposed drugs that were used clinically against other diseases [23]. These drugs have severe side effects including hepatoxicity, and thus high concentrations are needed. In this study, we have evaluated a range of laboratory and clinically-used compounds including metformin, quinclorac, indaziflam, inositol, nateglinide, 2,6-DNBT, trans-cinnamic acid, terbuthylazine, acarbose, glimepiride, vildagliptin, cellulase, thaxtomin A, repaglinide and dimethyl peptidase (IV) inhibitor to determine their effects against *N. fowleri* and *B. mandrillaris* cells. 

### 3.1. Selected Compounds Exhibited Potent Amoebicidal Activities against N. fowleri

**Figure 1:**
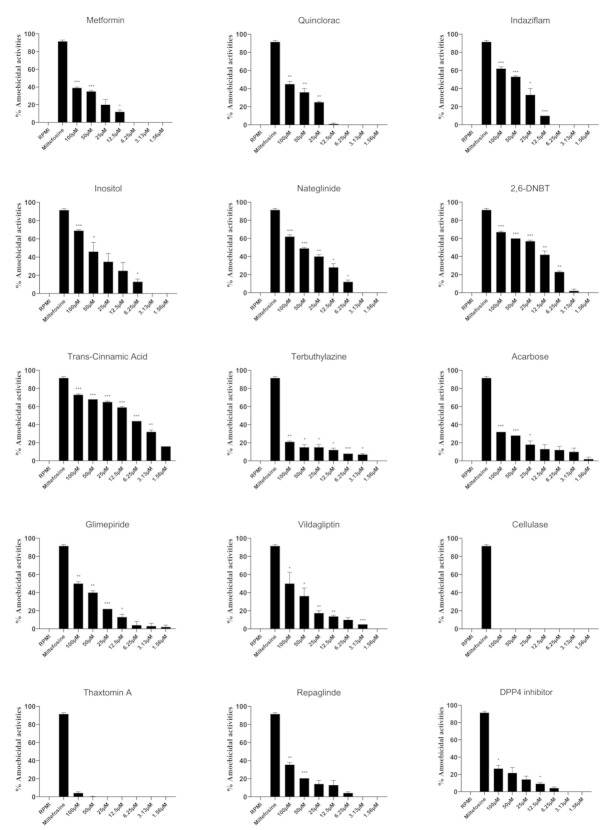
The amoebicidal activities of compounds against *N. fowleri* trophozoites. *N. fowleri* trophozoites were incubated with various concentrations of compounds for 24 h and trophozoites viability was determined using Trypan blue. Percentage amoebicidal activity was calculated by: [((RPMI cell count − cell count for sample)/RPMI cell count) × 100)]. The activities of solvents alone were negated from the activities of the compounds. The data is presented as the mean ± standard error (*: *p* < 0.05, **: *p* < 0.01, ***: *p* < 0.001 using student *t*-test; two tailed distribution).

Amoebicidal activities of compounds against *N. fowleri* were investigated at a range of concentrations (Figure 1). The half maximal effective concentration (EC_50_) of the compounds were determined as shown in Table 2, and it was found that acarbose, glimepiride, terbuthylazine and trans-cinnamic acid showed the best activities with EC_50_ values of 3.6 μM, 5.2 μM, 7.5 μM and 9.4 μM, respectively (Table 2). Also, repaglinide, dimethyl peptidase (IV) inhibitor and 2,6-DNBT had EC_50_ values of 14.3 μM, 14.9 μM and 24.2 μM, respectively (Table 2). When compared to solvent controls, trans-cinnamic acid, terbuthylazine and vildagliptin exhibited significant amoebicidal effects at 3.13 µM and above (*p* < 0.05), while inositol, nateglinide and 2,6-DNBT showed significant amoebicidal activities at 6.25 µM and above (*p* < 0.05). The lowest concentration at which metformin, indaziflam, glimepiride and DPP-4 inhibitor showed significant amoebicidal activities was 12.5 µM. Quinclorac and acarbose showed anti-amoebic activities at 25 µM and higher while repaglinide only showed activities at 50 µM and 100 µM. In contrast, cellulase and thaxtomin A did not show anti-*N. fowleri* effects at any concentration tested, when compared to the solvent control.

### 3.2. Selected Compounds Showed Anti-B. mandrillaris Properties

**Figure 2:**
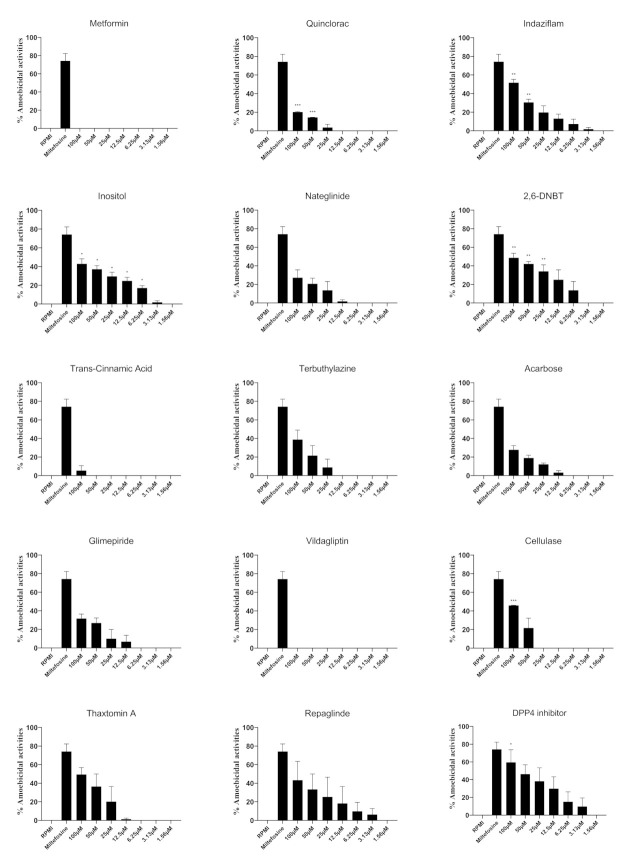
The amoebicidal activities of compounds against *B. mandrillaris* trophozoites. *B. mandrillaris* trophozoites were incubated with various concentrations of compounds for 24 h and trophozoites viability was determined using Trypan blue. Percentage amoebicidal activity was calculated by: [((RPMI cell count − cell count for sample)/RPMI cell count) × 100)]. The activities of solvents alone were negated from the activities of the compounds. The data is presented as the mean ± standard error (*: *p* < 0.05, **: *p* < 0.01, ***: *p* < 0.001 using student *t*-test; two tailed distribution).

Amoebicidal activities of compounds at a range of concentrations against *B. mandrillaris* were investigated (Figure 2). Table 2 depicts the EC_50_ values of dimethyl peptidase (IV) inhibitor, repaglinide, thaxtomin A and 2,6-DNBT was found to be 13.8 μM, 31.8 μM, 39.0 μM and 39.6 μM, respectively. As compared to solvent controls, among the several compounds tested, inositol and 2,6-DNBT showed significant amoebicidal activities against *B. mandrillaris* at 6.25 µM and 25 µM respectively. At 50 µM, quinclorac and indizaflam showed amoebicidal activities while cellulase and DPP-4 inhibitor showed anti-amoebic activities at 100 µM. None of the other compounds tested showed amoebicidal effects against *B. mandrillaris* when compared to the solvent control. 

### 3.3. Excystation of N. fowleri and B. mandrillaris was Inhibited by Selected Compounds

**Figure 3:**
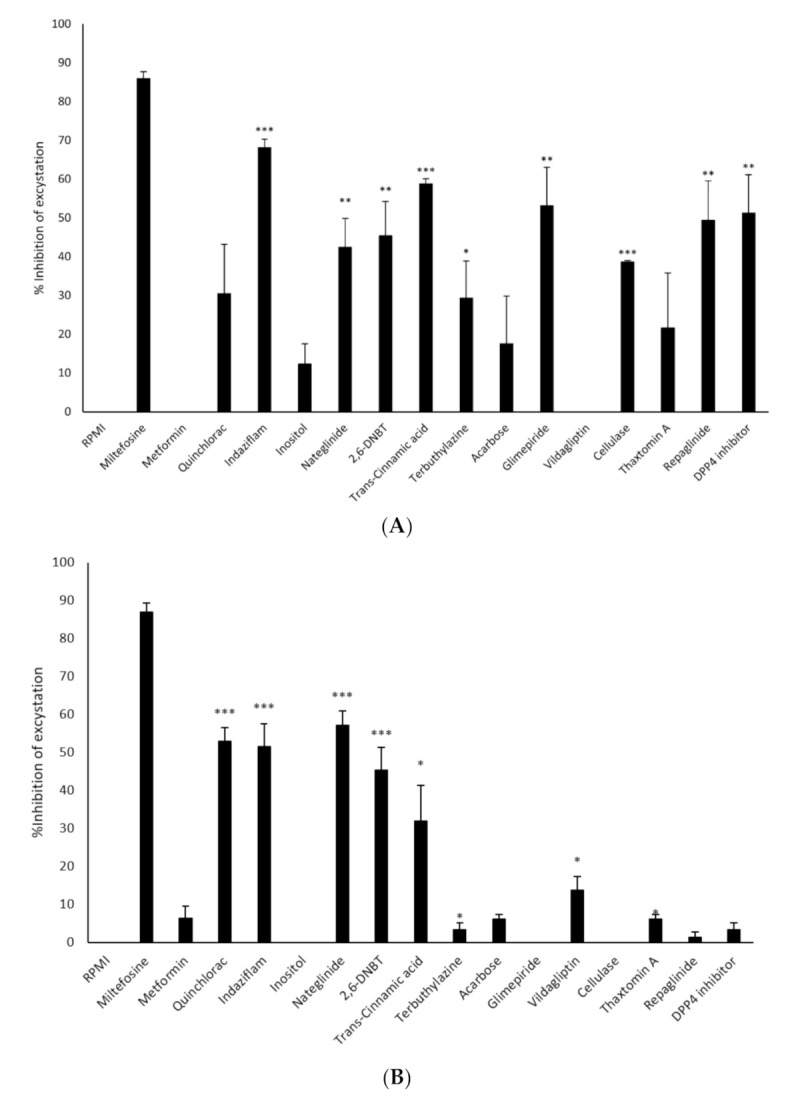
(**A**) Inhibition of excystation of *N. fowleri* cysts. *N. fowleri* cysts were incubated with 100 μM drugs and/or solvent in 24-well plates containing RPMI-1640 on HeLa monolayers for 24 h and number of viable trophozoites were then enumerated. Percentage inhibition of excystation was calculated by: [((RPMI trophozoite cell count − trophozoite cell count for sample)/RPMI trophozoite cell count) × 100)]. The activities of solvents alone were negated from the activities of the compounds. The data is presented as the mean ± standard error (*: *p* ˂ 0.05, **: *p* < 0.01, ***: *p* < 0.001 using student *t*-test; two tailed distribution). (**B**) Inhibition of excystation of *B. mandrillaris* cysts. *B. mandrillaris* cysts were incubated with 100 μM drugs and/or solvent in 24-well plates containing RPMI-1640 on HeLa monolayers for 24 h and number of viable trophozoites were then enumerated. Percentage inhibition of excystation was calculated by: [((RPMI trophozoite cell count − trophozoite cell count for sample)/RPMI trophozoite cell count) × 100)]. The activities of solvents alone were negated from the activities of the compounds. The data is presented as the mean ± standard error (*: *p* ˂ 0.05, **: *p* < 0.01, ***: *p* < 0.001 using student *t*-test; two tailed distribution).

The ability of the compounds to inhibit the excystation of *N. fowleri* cysts was investigated (Figure 3A). The results revealed that indaziflam, trans-cinnamic acid and glimepiride inhibited excystation of *N. fowleri* by 68%, 59% and 53% while DPP-4 inhibitor and repaglinide showed 51% and 49% inhibition, respectively after negating effects of solvent alone. 45% and 42% inhibition of excystation were observed for 2,6-DNBT and nateglinide, while cellulase and terbuthylazine caused 39% and 29% inhibition of excystation, respectively after negating effects of solvent alone. Although quinclorac, thaxtomin A, acarbose and inositol resulted in 30%, 22%, 17% and 12% inhibition of excystation, respectively, the activities were not significant as compared to respective solvent control. Also, metformin and vildagliptin did not inhibit excystation of *N. fowleri* (Figure 3A). 

In contrast, nateglinide and quinclorac resulted in 57% and 53% inhibition of excystation of *B. mandrillaris* while indaziflam, 2,6-DNBT, trans-cinnamic acid and vildagliptin inhibited excystation by 52%, 45%, 32% and 14%, respectively after negating effects of solvent alone (Figure 3B). Thaxtomin A, terbuthylazine, acarbose, DPP-4 inhibitor, repaglinide and glimepiride caused less than 10% inhibition of the excystation of *B. mandrillaris* cysts after negating effects of solvents alone (Figure 3B).

### 3.4. All of the Compounds Tested Exhibited Minimal Cytotoxicity against Human Epithelial Cells

**Figure 4:**
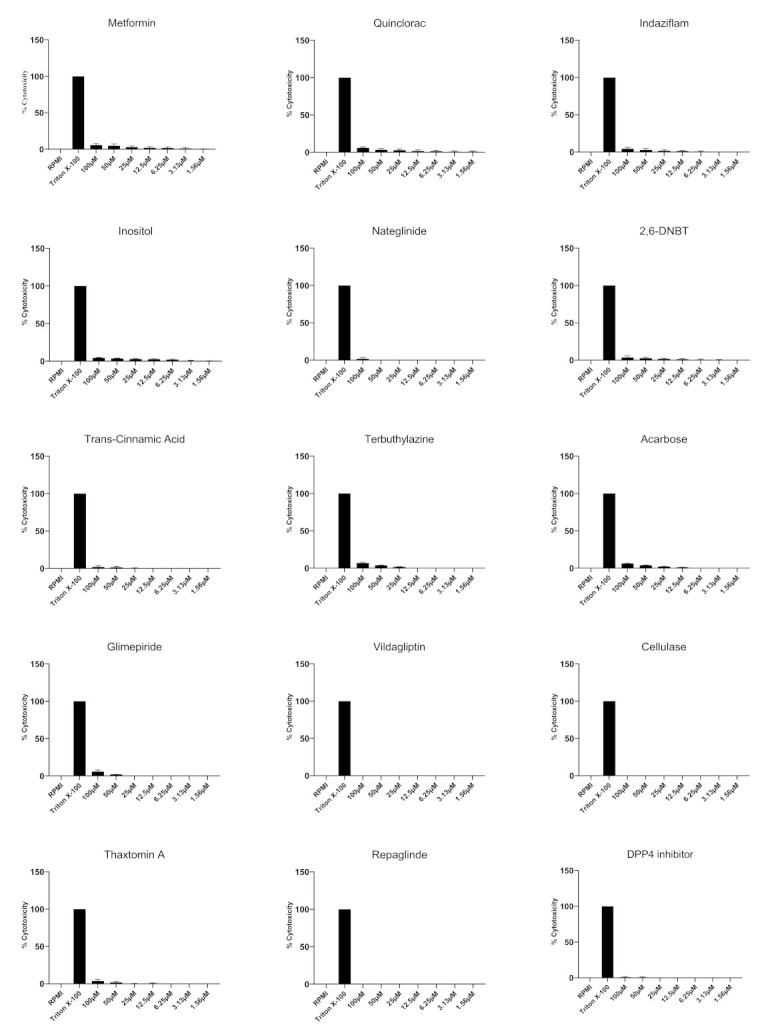
Cytotoxic effects of compounds against human cells are depicted. Various concentrations of compounds were incubated with human cells for 24 h at 37 °C and 5% CO_2_. RPMI alone was used as negative control and triton X-100 was used as positive control. Data are presented as the mean ± standard error of 2 experiments conducted in duplicates.

Cytotoxic activities of compounds against HaCaT cells were investigated at a range of concentrations. None of the compounds tested showed any significant damage to human cells as determined by LDH release (Figure 4). For example, vildagliptin, cellulase and repaglinide did not exhibit any cytotoxicity against HaCaT cells. DPP-4 inhibitor showed 1% cytotoxic activity while nateglinide and terbuthylazine revealed 2% activity. While 2,6-DNBT expressed 3% cytotoxicity, indazilfam, inositol and thaxtomin A exhibited 4% cytotoxicity. Metformin, quinclorac, acarbose and glimepiride caused 6% cytotoxicity whilst trans-cinnamic acid caused 8% cytotoxic activity.

Of note, 6 compounds, namely indaziflam, nateglinide, 2,6-DNBT, terbuthylazine, acarbose and glimepiride showed amoebicidal effects and inhibited excystation of both *N. fowleri* and *B. mandrillaris*. Indaziflam exhibited 68% and 52% inhibition of excystation and 62% and 52% amoebicidal activities against *N. fowleri* and *B. mandrillaris*, respectively. Nateglinide showed 42% and 53% inhibition of excystation and 64% and 39% amoebicidal activities. Similarly, 2,6-DNBT, terbuthylazine, acarbose, glimepiride exhibited 45%, 69%, 57%, 93% inhibition of excystation of *N. fowleri* cysts; and inhibition of excystation of *B. mandrillaris* cysts by 52%, 63%, 51% and 38%. Notably, 2,6-DNBT, terbuthylazine, acarbose, glimepiride exhibited 69%, 63%, 74% and 92% amoebicidal activities against *N. fowleri* and 60%, 61%, 60% and 54% anti-amoebic activities against *B. mandrillaris*.

Nateglinide, acarbose and glimepiride are FDA approved anti-diabetic drugs that are commonly used for clinical application, thus their pharmacological and safety profiles have already been evaluated [26,27,28,29]. This is especially useful, as phase II clinical trials are not possible due to the rarity and fatality caused by the diseases due to free-living amoebae, with the only remaining option to evaluate drug efficacy using in vitro and in vivo techniques [30]. Thus, the findings from our study are encouraging and further affirm the concept of repurposing drugs for use against infections due to brain-eating amoebae. Considering that the aforementioned compounds showed amoebicidal effects as well as inhibited excystation for both *N. fowleri* and *B. mandrillaris* when tested individually, effects of the compounds when used in combination needs to be evaluated. Also, the mechanism of actions of drugs against *N. fowleri* and *B. mandrillaris* are yet to be determined and need to be assessed in future work. It is likely that, as glucose is an important constituent of the amoebae cyst wall and is important in cyst wall biosynthesis, these drugs may be targeting glucose, however this needs to be investigated.

In addition, these repurposed compounds should be tested in the novel intranasal route to treat infections due to brain eating amoebae, which has been proposed in recent years. This route may have many advantages, as the existing compounds used to eliminate amoebae are unable to cross the blood-brain barrier (BBB) and result in hepatoxicity in patients at the doses needed to eradicate the CNS residing amoebae [31,32]. Furthermore, previous studies have revealed that intranasal delivery of amphotericin B was shown to be effective in the treatment of invasive mucormycosis [33]. Thus, it will be necessary to prepare vaporised formulations of these repurposed compounds and evaluate their efficacy intranasally, in addition to intravenous efficacy evaluation in vivo. 

## 4. Conclusions

To our knowledge, for the first time, we demonstrated that indaziflam, nateglinide, 2,6-DNBT, terbuthylazine, acarbose and glimepiride possess amoebicidal properties and inhibit excystation of *N. fowleri* and *B. mandrillaris* suggesting the potential of these compounds as anti-amoebic agents. However, future studies are needed to determine the translational value of these findings individually and in combination against *N. fowleri* and *B. mandrillaris* cells in vivo.

## Figures and Tables

**Table 1:** Chemical nomenclature, empirical formula, class, mechanism of action, molar mass and solvent solubility of compounds evaluated against *N. fowleri* and *B.* mandrillaris [24,25,26].

Compound	Trade Name	Emperical Formula	IUPAC Name	Molar Mass	Mechanism of Action	BBB Permeability	Solvent
Metformin	Glucophage, Riomet, Fortamet, etc.	C_4_H_11_N_5_	1-carbamimidamido-N,N-dimethylmethanimidamide	165.63	Protein kinase activity inducer; Ubiquinone binding inhibitor; NAD binding inhibitor	Positive	RPMI-1640
Quinclorac	Not used clinically	C_10_H_5_C_l2_NO_2_	3,7-Dichloro-8-quinolinecarboxylic acid	242.06	Acetylcholinesterase inihbitor; binds to albumin	Not available	RPMI-1640
Indaziflam	Not used clinically	C_16_H_20_FN_5_	N-(2,3-dihydro-2,6-dimethyl-1H-inden-1-yl)-6-(1-fluoroethyl)-1,3,5-triazine-2,4-diamine	301.36	Cellulose biosynthesis inhibitor	Not available	RPMI-1640
Inositol	Ovasitol, Inositech, Niacinol, etc.	C_6_H_12_O_6_	cyclohexane-1,2,3,4,5,6-hexol	180.16	Phosphoric diester hydrolase activity; Glucosylceramidase receptor binding; Manganese ion binding inhibitor	Present in brain	RPMI-1640
Nateglinide	Starlix	C_19_H_27_NO_3_	(2R)-3-phenyl-2-[(4-propan-2-ylcyclohexanecarbonyl)amino]propanoic acid	317.43	Sulfonylurea receptor inhibitor; Zinc ion binding agonist	Positive	20% Ethanol
Dichlobenil (2,6-DNBT)	Not used clinically	C_7_H3Cl_2_N	2,6-dichlorobenzonitrile	172.01	Cellulose synthesis inhibitor	Not available	20% Ethanol
Trans-cinnamic acid	Not used clinically	C_9_H_8_O_2_	(E)-3-phenylprop-2-enoic acid	148.16	Hydroxycarboxylic acid receptors agonist	Not available	20% Ethanol
Terbuthylazine	Not used clinically	C_9_H_16_ClN_5_	2-N-tert-butyl-6-chloro-4-N-ethyl-1,3,5-triazine-2,4-diamine	229.71	Triazine selective systemic herbicide affecting electron transport	Not available	20% DMSO
Acarbose	Precose	C_25_H_43_NO_18_	(3R,4R,5S,6R)-5-[(2R,3R,4R,5S,6R)-5-[(2R,3R,4S,5S,6R)-3,4-dihydroxy-6-methyl-5-[[(1S,4R,5S,6S)-4,5,6-trihydroxy-3-(hydroxymethyl)cyclohex-2-en-1-yl]amino]oxan-2-yl]oxy-3,4-dihydroxy-6-(hydroxymethyl)oxan-2-yl]oxy-6-(hydroxymethyl)oxane-2,3,4-triol	645.61	alpha-glucosidases inhibitor	Negative	20% DMSO
Glimepiride	Amaryl	C_24_H_34_N_4_O_5_S	4-ethyl-3-methyl-N-[2-[4-[(4-methylcyclohexyl)carbamoylsulfamoyl]phenyl]ethyl]-5-oxo-2H-pyrrole-1-carboxamide	490.62	Voltage-gated potassium channel inhibitor; Phosphatidylinositol-4,5-bisphosphate binding inhibitor; Sulfonylurea receptor inducer	Positive	20% DMSO
Vildagliptin	Galvus	C_17_H_25_N_3_O_2_	(2S)-1-[2-[(3-hydroxy-1-adamantyl)amino]acetyl]pyrrolidine-2-carbonitrile	303.4	Dipeptidyl peptidase 4 inhibitor	Positive	RPMI-1640
Cellulase	Not used clinically	C_18_H_32_O_16_	(2S,3R,4S,5S,6R)-2-[(2R,3S,4R,5R,6S)-4,5-dihydroxy-2-(hydroxymethyl)-6-[(2R,3S,4R,5R,6R)-4,5,6-trihydroxy-2-(hydroxymethyl)oxan-3-yl]oxyoxan-3-yl]oxy-6-(hydroxymethyl)oxane-3,4,5-triol	504.4	Hydrolysis of 1,4-beta-glucosidic linkages in cellulose	Not available	RPMI-1640
Thaxtomin A	Supplied by Adipogen as antibiotic	C_22_H_22_N_4_O_6_	(3R,6S)-3-hydroxy-3-[(3-hydroxyphenyl)methyl]-1,4-dimethyl-6-[(4-nitro-1H-indol-3-yl)methyl]piperazine-2,5-dione	483.43	Not Available	Not available	20% DMSO
Repaglinide	Prandin, NovoNorm, Enyglid, etc.	C_27_H_36_N_2_O_4_	2-ethoxy-4-[2-[[(1S)-3-methyl-1-(2-piperidin-1-ylphenyl)butyl]amino]-2-oxoethyl]benzoic acid	452.59	Potassium voltage-gated channel inhibitor; Sulfonylurea receptor activity inhibitor; Zinc ion binding agonist	Negative	20% DMSO
dimethyl peptidase (IV) inhibitor	Januvia, Onglyza, Tradjenta, etc.	Not Available	Not Available	328.41	Dipeptidyl peptidase 4 inhibitor	Negative	20% DMSO

**Table 2:** Half maximal effective concentration (EC_50_) of the compounds against *N. fowleri* and *B. mandrillaris*.

Compound	EC_50_ (μM)	
	*N. fowleri*	*B. mandrillaris*
Metformin	134.0	Not available
Quinclorac	102.1	296.6
Indaziflam	53.7	99.8
Inositol	48.9	124.1
Nateglinide	44.4	136.2
Dichlobenil (2,6-DNBT)	24.2	39.6
Trans-cinnamic acid	9.4	Not available
Terbuthylazine	7.5	62.5
Acarbose	3.6	80.8
Glimepiride	5.2	63.6
Vildagliptin	100.3	Not available
Cellulase	Not available	106.0
Thaxtomin A	94.7	39.0
Repaglinide	14.3	31.8
dimethyl peptidase (IV) inhibitor	14.9	13.8

## Data Availability

Not applicable.
